# A Critical Review on Misleading Evidence in Cardiac Arrest Trials—Why Less Complexity Does Not Result in Better Outcomes

**DOI:** 10.3390/jcm15020821

**Published:** 2026-01-20

**Authors:** Andreas Schäfer, Tobias J. Pfeffer, Johann Bauersachs, Vera Garcheva

**Affiliations:** Cardiac Arrest Centre, Department of Cardiology and Angiology, Hannover Medical School, D-30625 Hannover, Germany; pfeffer.tobias.j@mh-hannover.de (T.J.P.); bauersachs.johann@mh-hannover.de (J.B.); garcheva.vera@mh-hannover.de (V.G.)

**Keywords:** out-of-hospital cardiac arrest, therapeutic hypothermia, supraglottic airway, post-resuscitation care, Hannover Cardiac Resuscitation Algorithm (HaCRA)

## Abstract

Over the past two decades, advanced airway management, early coronary angiography, and therapeutic hypothermia have shaped post-out-of-hospital cardiac arrest (OHCA) care. However, recent large randomized trials have challenged these strategies and created substantial uncertainty leading to relevant guideline changes. This review focuses on the trials that ultimately influenced current guideline recommendations by downgrading previous recommendations. We determine how structural limitations may have affected the validity and interpretation of their results. The review critically evaluates the methodological design and execution of those trials. Despite neutral findings from recent randomized trials, use of advanced airway management during resuscitation, coronary angiography in patients with a high likelihood of acute coronary occlusion, and therapeutic hypothermia for comatose OHCA survivors still play a relevant role in post-resuscitation management.

## 1. Introduction

During ongoing resuscitation, paramedics traditionally used bag–valve–mask ventilation to provide oxygenation, while endotracheal intubation was applied to secure the airway, ensure adequate oxygenation and decarboxylation, and protect against aspiration. However, two large randomized controlled trials (RCTs) challenged this paradigm by reporting non-inferiority of easily placed laryngeal tubes or specific laryngeal masks (i-gel) compared with endotracheal intubation [1,2].

Following return of spontaneous circulation (ROSC), the first 24 h are critical for reperfusion. Despite restored oxygenation and circulation, a secondary wave of neuronal injury—reperfusion injury—occurs. Post-resuscitation care changed profoundly after two landmark RCTs demonstrated reduced mortality and improved neurological outcomes with early therapeutic hypothermia [3,4]. As control groups in these early trials were not strictly maintained normothermic and often developed fever, larger RCTs were later conducted to test the non-inferiority of temperature control at 36 °C [5] and, subsequently, controlled normothermia alone [6].

Because myocardial infarction is a major cause of cardiac arrest, early coronary angiography in ROSC patients without an obvious non-coronary cause of arrest was widely recommended to interrupt ongoing ischemia and prevent arrhythmias or heart failure. Although numerous registries supported this strategy [7,8,9], larger RCTs failed to show a mortality benefit [10,11]. While research results are limited in the field of resuscitation in general and even the right dose of adrenaline or any other potential vasopressor is not finally determined [12], we wanted to focus on those changes in recommendations where trials with per-protocol or by-conduct biases seemed to produce a non-inferior result resulting in de-escalation of guideline recommendations.

Across all three therapeutic domains, the consequences were similar: current resuscitation guidelines now recommend early placement of supraglottic airways, permissive hypothermia rather than fixed-target therapeutic hypothermia, and deferred coronary angiography to a later time in the hospitalization course [13,14]. Early reports regarding changes in reference to the new guideline recommendations, however, indicated a higher mortality rate following laryngeal tubing [15,16,17] and avoidance of therapeutic hypothermia [18,19,20]. Just to provide the magnitude of effect, in Germany, applying therapeutic hypothermia to all STEMI OHCAs would save 1000 lives per year [21]. Hence, in this article, we analyze the major RCTs in detail and highlight structural limitations that may have masked clinically relevant advantages of previous standards—potentially leading to guideline changes that do not fully reflect best clinical practice. In some of the trials, there is a common misinterpretation in the way that the absence of evidence is interpreted as the evidence of absence. However, this anticipation is often not correct, in the same way that a trial’s hypothesis is not automatically true because it has not been proven false. The lack of evidence can simply mean that a thorough investigation has not yet occurred. This can occur by (1) incorporating too many pre-hospital deaths, precluding any analysis of functional outcomes in survivors; (2) increasing the event rate in both groups, e.g., following withdrawal of life-sustaining therapy in the absence of negative prognostication; (3) including a high proportion of patients unlikely to benefit from an intervention; or either (4) delaying the randomized intervention to a point in time when it is ineffective or (5) omitting it at all.

## 2. Methods

We screened the most recent guidelines of the European Resuscitation Council (ERC) and the European Society of Intensive Care Medicine (ESICM) on adult cardiac life support and post-resuscitation care regarding strong changes in recommendations in 2021 [22,23] and 2025 [13,14] compared to previous versions, which were based on large neutral RCTs. We identified three topics (supraglottic airway devices, deferred coronary angiography in suspected cardiac cause of arrest, and fever prevention instead of therapeutic hypothermia) that resulted in de-escalated treatment based on neutral large RCTs. The publications of the six underlying RCTs, including supplementals or separately published protocols, were reviewed. These were screened for the presence of one or more of the five biasing conditions potentially interfering with neutrality. The trials under scrutiny, which were selected because of their effect on changes in guideline recommendations limiting post-arrest care, were checked in detail. The focus of our analysis was to identify either protocol recommendations or deviations that would significantly impact the validity of reported data in a way that precluded detection of potential differences in outcomes.

In general, neutral findings must be interpreted in the context of adherence and timing, and this requires distinguishing intention-to-treat from per-protocol or as-treated analyses. While intention-to-treat preserves the benefits of randomization and guards against selection bias, it may dilute true treatment effects when protocol adherence is low or when the intervention is not delivered within a biologically meaningful time window. Per-protocol or as-treated analyses may better reflect the effect of the intervention when actually received, particularly for time-sensitive therapies, but they introduce risks of confounding and selection bias. Therefore, such analyses should be interpreted as supportive or hypothesis-generating rather than confirmatory. In this context, neutral intention-to-treat results do not exclude potential benefit when the intervention is applied early and consistently, but alternative analyses must be weighed against their inherent bias trade-offs. While we cannot re-calculate positive findings from the published neutral data if the aforementioned affects had not happened, we like to generate some curiosity to question the impact of such effects on these neutral trials.

The research for this article was not supported by generative artificial intelligence.

## 3. A Critical View on the Trials

### 3.1. Advanced Airway Management During Resuscitation

Historically, endotracheal intubation performed by skilled personnel was long considered the gold standard for securing the airway during cardiac arrest, ensuring adequate oxygenation, ventilation, and protection from aspiration. Over the last 20 years, supraglottic airway devices—particularly laryngeal tubes and i-gel devices—have sequentially emerged as simpler alternatives, especially for less experienced rescuers [24,25]. Two major pre-hospital RCTs—one from the US evaluating laryngeal tubes [1] and one from the UK using the i-gel^®^ device [2]— suggested non-inferiority of supraglottic devices when compared with endotracheal intubation in OHCA patients (Table 1).

As shown in Table 1, both RCTs assessed the non-inferiority of supraglottic airway devices compared with endotracheal intubation in OHCA patients. Both trials together enrolled more than 12,000 OHCA patients, providing a strong and large cohort for analysis. However, they had several limitations: both trials included large proportions of unwitnessed arrests, low bystander CPR rates, and relatively few patients with shockable rhythms—conditions associated with extensive anoxic brain injury even before EMS arrival. While reflecting a representative real-world mix of OHCA situations, this constellation indicates a high likelihood of profound anoxic injury even before airway management, which might preclude analysis of long-term safety or non-inferiority in survivors. Consequently, both trials reported very low hospital admission and discharge rates, as well as low frequencies of favorable neurological outcomes. Given the small number of patients with potential for good neurological recovery, both studies carry a substantial risk of failing to detect clinically relevant inferiority in neurological outcomes.

This issue is further exacerbated by the high rate of failed attempts to establish the assigned airway strategy. Differences in failure rates between groups may reflect the participating sites’ training and proficiency rather than true equivalence or non-inferiority of the devices. Considering these limitations, both trials inherit a potential strong bias regarding the non-inferiority of supraglottic airway management compared with endotracheal intubation.

High primary success rates for endotracheal intubation (98%) and laryngeal tube exchange (89%) can be achieved even by non-anesthetists after short-term training using video laryngoscopy, as demonstrated in a non-academic emergency department in Germany [26]. Thus, physician-staffed emergency services—such as those in Germany, where both anesthetists and non-anesthetists respond to emergencies—can achieve higher intubation success rates than those reported in paramedic-based trials.

Endotracheal intubation is superior to supraglottic airway management during automated chest compression CPR [27]. To evaluate whether laryngeal tubes may increase the risk of anoxic brain injury due to inadequate oxygenation during resuscitation, we analyzed a decade of OHCA cases with presumed cardiac etiology and shockable rhythms—thereby minimizing anoxic causes of arrest. Patients ventilated with a laryngeal tube had a higher likelihood of anoxic brain damage compared to those receiving endotracheal intubation [15]. This finding aligns with several registry analyses from Germany and Austria reporting worse outcomes in laryngeal tube-treated patients compared with those receiving endotracheal intubation [16,17].

Even among supraglottic devices, meaningful differences exist. The i-gel has recently been favored over the laryngeal tube due to higher placement success and improved ventilation [28]. Nevertheless, endotracheal intubation remains the most reliable airway management strategy [28,29]. Training efforts should therefore refocus on enabling healthcare providers—both emergency physicians and paramedics—to competently perform endotracheal intubation in OHCA patients, using the i-gel as a backup device while avoiding laryngeal tubes whenever possible.

### 3.2. Early Coronary Angiography in OHCA Patients with Suspected Coronary Cause of Arrest

The 2021 post-resuscitation care guidelines suggested coronary angiography as the first-line approach in patients with myocardial ischemia and recommended a strong consideration of immediate coronary angiography in OHCA without ST-elevation if coronary occlusion was considered highly probable [22]. In contrast, the 2025 guidelines downgraded and narrowed this recommendation to patients with ST-elevation and a delayed catheterization strategy for those without ST-elevation [14].

The earlier recommendation was clinically plausible and based on retrospective analyses and small observational studies. The rationale was straightforward: myocardial infarction is a leading cause of cardiac arrest in otherwise healthy individuals without an alternative explanation for collapse [30,31]. Since cardiac arrest represents the most severe complication of myocardial infarction, it seemed reasonable to promptly clarify the coronary anatomy in patients without an obvious non-cardiac cause and with suspected ischemia. Registries of OHCA patients showed that early coronary angiography after ROSC was associated with higher survival and better neurological outcomes—even in the absence of ST-segment elevation [7,8,9]. However, two prospective RCTs challenged these assumptions: one enrolling only shockable OHCA patients without ST-elevation (COACT) [10] and the other including all non-ST-elevation OHCA cases (TOMAHAWK) [11] (Table 2). Both trials suggested that a deferred angiographic strategy—though counterintuitive—did not worsen clinical outcomes. Hence, the current guidelines chose a more reluctant recommendation regarding early coronary angiography in OHCA patients without ST-segment elevations [14].

Despite both trials together comprising more than 1000 OHCA patients, which allows for a good patient volume to assess, several limitations of COACT [10] and TOMAHAWK [11] deserve attention. First, both trials broadly enrolled so-called NSTE-OHCA patients, but low revascularization rates suggest suboptimal pre-selection for true acute coronary occlusion. A considerable fraction had previous myocardial infarction, making an arrhythmic cause of arrest plausible. Second, in TOMAHAWK, every second patient had a non-shockable rhythm, further lowering the probability of acute coronary ischemia. Third, low bystander CPR rates and consequently prolonged no-flow times may have contributed to high mortality in both groups due to extensive anoxic brain injury. Consequently, the trials may not have been able to detect benefit even if present. Subsequent guideline updates have therefore adopted a more restrictive stance, recommending immediate angiography only in patients with ST-elevation, which may overlook patients with subtle or atypical presentations.

**Figure 1:**
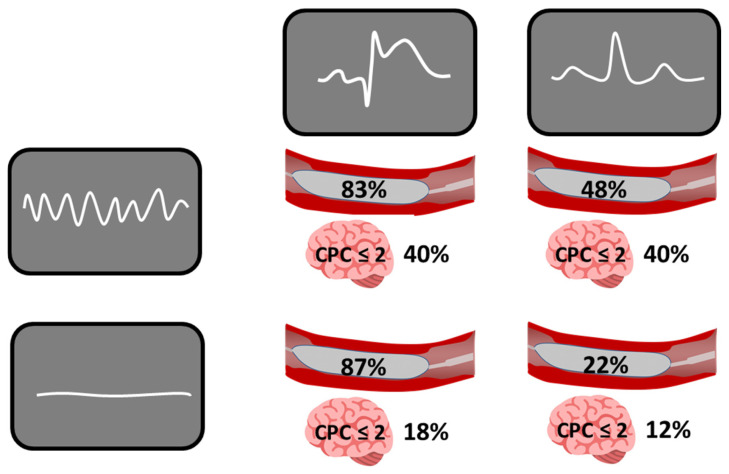
Influence of shockable and non-shockable rhythms and presence or absence of ST-segment elevation in out-of-hospital cardiac arrest patients with return of spontaneous circulation to predict the presence of relevant coronary stenosis (depicted as the inflated balloon within a coronary stenosis) and favorable neurological outcomes (defined by a cerebral performance category (CPC) score of 2 or less) (modified according to Garcheva V et al. [33].

Today, improved pre-selection is essential to identify patients with a high probability of ongoing coronary ischemia. Computed tomography—which should be part of the admission work-up—may support this process. Notably, in OHCA cohorts requiring revascularization, more complete and extensive revascularization has been independently associated with improved outcomes [32]. Overall, identifying the right patient (ongoing coronary ischemia) for the right treatment (coronary angiography and intervention) is crucial. In our OHCA population, among the two most relevant ECG features (shockable vs. non-shockable rhythm and presence vs. absence of ST-segment elevation), ST-segment elevation best predicted coronary stenosis, whereas a shockable rhythm better predicted favorable neurological outcomes (Figure 1).

**Figure 2:**
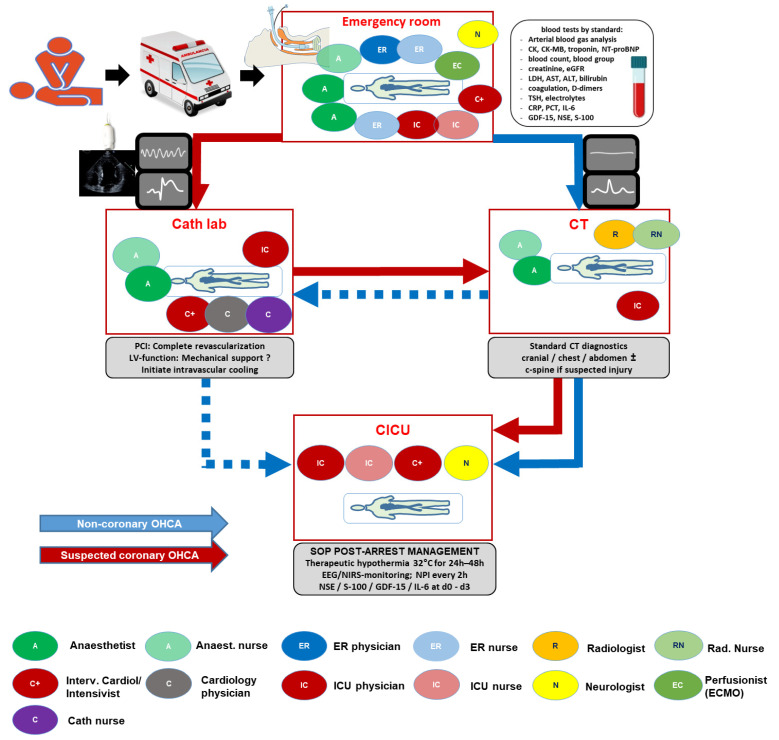
An interdisciplinary algorithm such as the Hannover Cardiac Resuscitation Algorithm (HaCRA) is a treatment algorithm delineating diagnostic and interventional responsibilities of all participating professions (emergency room, cardiology, anesthesiology, radiology, neurology, intensive care) in the work-up of any out-of-hospital cardiac arrest or cardiogenic shock patient. Adopted from Garcheva V et al. [39]. CICU—cardiology intensive care unit; CT—computed tomography; EEG—electro-encephalogram; ER—emergency room; GDF-15—growth-derived factor 15; IL-6—interleukin 6; NIRS—near-infrared spectroscopy for cerebral oxygenation; NPI—neurological pupil index; NSE—neuron-specific enolase; PCI—percutaneous coronary intervention; S-100—protein S-100.

Therefore, it appears reasonable to select patients with potential for good neurological outcomes based on basic parameters and to offer coronary angiography particularly to those with a shockable rhythm and no alternative cause of arrest [34,35,36,37,38]. Computed tomography imaging at admission—eventually supported by artificial intelligence interpretation—may help identify reversible coronary causes. Among resuscitated patients who ultimately undergo revascularization, more complete revascularization independently predicts improved outcomes, underscoring that the challenge is identifying the right patient for early coronary intervention. The incorporation of coronary catheterization and computed tomography in a standard operating procedures is illustrated in Figure 2.

### 3.3. Therapeutic Hypothermia

The introduction of therapeutic hypothermia in the early 2000s revolutionized post-ROSC care. In 2002, two landmark RCTs demonstrated substantial reductions in mortality and poor neurological outcomes in comatose OHCA patients treated with 32–34 °C. Therapeutic hypothermia in OHCA patients reduced mortality by 25% and poor neurological outcomes by 27% [3,4,40]. Although these studies would today be considered underpowered or pilot trials, they reflected the clinical research standards of their time. Their findings have since been consistently confirmed, and more recent meta-analyses still show a benefit of hypothermia in OHCA survivors [40,41]. However, because the control groups did not receive an active temperature intervention—and therefore were not strictly maintained normothermic [3,4]—and post-arrest fever is associated with worse outcomes [42], it remained unclear whether the observed effects were due to hypothermia in the intervention group or due to fever in the controls [5]. Hence, two targeted temperature management (TTM trials were conducted to test the hypothesis of whether either a targeted temperature of 36 °C or controlled normothermia is non-inferior to hypothermia (Table 3).

Together, both TTM trials enrolled 2800 OHCA patients, providing sufficiently large cohorts for useful statistical analysis. While being adequately powered to address the hypotheses in terms of size and design, both trials had several other limitations.

In 2013, the TTM1 trial compared hypothermia at 33 °C with temperature management at 36 °C and created substantial uncertainty regarding the necessity of hypothermia. TTM1 enrolled 950 OHCA patients—far larger and more rigorously conducted than the earlier trials—yet found no benefit of hypothermia, despite similarly high mortality rates of 48–50% [5]. A major limitation was the delay in achieving the target temperature: randomization took about 3 h, and patients assigned to hypothermia needed an additional 7 h on average to reach 33 °C. Therapeutic hypothermia requires reaching target temperature within 6 h after ROSC to minimize poor outcomes; thus, most patients in TTM1 failed to reach target temperature in time [43].

When analyzing only TTM1-like patients from the HAnnover COoling REgistry (HACORE)—where all patients underwent rapid and effective intravascular cooling according to protocol [39,44]—we found an in-hospital mortality of only 25%, compared with 44% in TTM1, despite similar patient characteristics and real-world clinical conditions [39,44]. Comparable, substantially lower mortality rates were also reported in several smaller contemporary studies involving TTM1-like patients [45,46].

Following TTM1 and guideline revisions recommending temperature control anywhere between 32 and 36 °C [22], many centers abandoned strict hypothermia. This shift correlated with worsening outcomes: the Melbourne group, which had actually conducted one of the historic landmark trials for hypothermia, changed their treatment according to the TTM1 protocol and compared cohorts before and after transitioning to TTM1-like normothermia. They found higher mortality and less favorable neurological outcomes with normothermia [18]. Data from more than 15,000 OHCA patients in Australia and New Zealand showed that declining post-arrest temperatures prior to TTM1 were associated with declining mortality, whereas rising temperatures following TTM1 publication were linked to increasing mortality [47]. Similar findings emerged from the UK in more than 30,000 OHCA patients: body temperature steadily fell prior to TTM1 and rose after publication, and mortality increased in parallel [19]. A Japanese multicenter analysis of 125 ICUs showed that patients with intermediate severity of post-cardiac arrest syndrome (excluding easy survivors and moribund patients) had better outcomes with hypothermia than normothermia. Overall, observational data consistently show that rising post-arrest temperatures parallel rising mortality; normothermia does not appear equivalent to targeted hypothermia at 33 °C [18,19,20,47,48].

The TTM2 trial, enrolling 1900 OHCA patients, added further confusion. Unlike TTM1, it aimed to compare hypothermia at 33 °C with fever prevention rather than normothermia. Again, results suggested equivalence [6]. However, the protocol required that target temperature be reached as soon as possible, ideally within 90 min—yet this was never achieved. Time from ROSC to randomization averaged 110 min, and median time to reach 33 °C was 5 h after randomization, meaning that half the patients reached target temperature about 7 h after ROSC [43]. Thus, as in TTM1, most patients in TTM2 did not receive therapeutic hypothermia within the critical 6 h window; on average, they required more than 7 h (TTM1: >9 h) [5,6].

A second major issue was the protocolized early withdrawal of life support. Although required by protocol, the number and timing of neurological assessments suggest that rigorous prognostication was unlikely. Consequently, mortality as an endpoint was influenced by early withdrawal of life support, creating a bias toward neutral outcomes and further obscuring any potential benefit of hypothermia. This uncertainty in prognostication was eventually influenced by lack of strict recommendations regarding prognostication in guidelines, which led to divergent approaches in later guideline recommendations and trial protocols. The differing criteria are now shown in Table 4. Overall, withdrawal of life-sustaining therapy based on neurological reasons occurred in 497 patients in both TTM trials; however, 194 (39%) of these cases did not fulfill two or more of the criteria demanded by current ERC/ESICM guideline recommendation for poor prognosis [49].

In summary, the methodologies of TTM1 and TTM2 did not adequately test the intended intervention hypotheses. In both trials, the majority of patients did not receive the intended hypothermia treatment within the required time window, and early withdrawal of life support introduced substantial confounding. Given these methodological limitations, the impact on guidelines by both trials and consecutive abundance of therapeutic hypothermia is debatable. It is of uttermost importance that a per-protocol analysis be performed for both trials, which will only include a minority of the trials’ overall population.

Real-world evidence and biological plausibility continue to support rapid induction of therapeutic hypothermia to 33 °C, especially in patients without devastating anoxic injury. Observational analyses from Australia and New Zealand, the UK, Germany, and Japan consistently show that population-level declines in post-arrest temperature correlate with improved outcomes, while shifts toward normothermia correlate with increased mortality [18,19,20,21,43,44,47].

## 4. Discussion and Future Options

Over the past two decades, post-arrest care has undergone substantial evolution, driven initially by promising results from early RCTs and later challenged by more recent, larger studies. This development has created significant uncertainty regarding optimal management strategies in three key domains: airway management during resuscitation, targeted temperature management after ROSC, and early coronary angiography in comatose survivors without ST-segment elevation (Table 5). A careful analysis of the available evidence, including methodological limitations of recent trials, suggests that several changes widely adopted in the guidelines [13,14] may not be fully supported by robust data.

When looking at the structural problems within the airway management trials, the low success rate in endotracheal intubation, which still is the gold standard, indicates a significant need for training and qualification of rescuers. Professional rescuers, e.g., paramedics, should be qualified to use video-laryngoscopy under resuscitation. How can we assume that the same rescuers will handle even more problematic airways, e.g., with severe bleeding in trauma patients, if they fail with a non-resisting body in cardiac arrest?

Regarding coronary angiography after cardiac arrest, both large trials demonstrate that current approaches to identify the likelihood of coronary thrombosis are not predictive enough. Considering the wider implementation of post-resuscitation whole-body computed tomography might open an opportunity for non-invasive coronary angiography, maybe supported by artificial intelligence. If such a strategy helps to reliably identify patients with relevant coronary stenosis non-invasively during admission work-up, only patients requiring PCI will be transported to the catheterization laboratory. This will massively enhance pre-selection and contribute to better resource allocations.

With respect to therapeutic hypothermia, there is still an unmet need for comatose OHCA survivors. None of the tools explored, such as predictive scores or imaging upon admission, is as yet reliable enough to discriminate between long-term survivors with good outcomes and futile patients. Again, here, artificial intelligence may eventually help us in the future to increase the accuracy of early prediction. In large hypothermia trials, neurological prognostication was performed from day 3 onwards; however, it has recently been reported that a large number of patients were deemed futile without meeting the required prognostication results suggested by ERC/ESICM guidelines. Hence, despite the majority of patients not even receiving hypothermia as demanded by the protocol, post hoc analyses suggest a meaningful proportion of neurological withdrawal of life-sustaining therapy occurred in patients with an indeterminate prognosis by conservative criteria, raising concern about a self-fulfilling prophecy [49]. Until we have better and earlier prediction tools, we might have to stick to the therapy demonstrating outcome benefits in the decades preceding the TTM trials.

## 5. Limitations

This critical review is focused on three topics in which large RCTs changed guideline practice based on presumed neutral efficacy by de-escalating therapy in post-resuscitation care. This approach leaves some limitations: rather than identifying the respective studies in large databases by a structured literature search, we focused on the large RCTs that actually induced the guideline changes. They were driven by reducing complexity, but eventually, background therapy was not defined solidly enough. Hence, this approach might have introduced a significant selection bias.

## 6. Conclusions

Methodological limitations of recent large pragmatic trials may have led to guideline changes that insufficiently reflect biological plausibility in post-cardiac arrest care. Persistent uncertainty remains regarding the non-inferiority of supraglottic airways—particularly the laryngeal tube—limiting temperature management to fever prevention and deferred coronary angiography. Considering diluted true treatment effects due to low protocol adherence not delivering hypothermia within a biologically meaningful time window may have relevantly impacted on the validity of large RCTs that led to changes in guideline recommendations. Hence, despite large ITTs, it may be reasonable to maintain advanced airway management, rapid therapeutic hypothermia, and early coronary angiography in appropriately selected OHCA patients within specialized cardiac arrest centers.

## Figures and Tables

**Table 1:** Patient characteristics in RCTs comparing supraglottic airway devices to endotracheal intubation in out-of-hospital cardiac arrest.

	Wang et al. [1]	AIRWAYS-2 [2]
Patients, n	3004	9296
Witnessed arrest, n (%)	1399 (47)	5889 (63)
Bystander CPR, n (%)	1407 (47)	5923 (64)
Shockable rhythm, n (%)	571 (19)	2156 (23)
Died at scene, n (%)	1209 (40)	5111 (55)
Died prior to ICU admission, n (%)	not reported	7395 (80)
Survival to discharge, n (%)	284 (9)	764 (8)
Primary outcome, days	3	30
CPC 1&2, n (%)	182 (6)	611 (7)
Median time to death, min	not reported	63/67
Primary airway failure		
in ETI group, n/N (%)	573/1299 (44)	2247/4410 (51)
in SGA group, n/N (%)	159/1353 (12)	1421/4886 (29)
Transport with assigned airway		
in ETI group, n/N (%)	1160 (77)	1008/1429 (71)
in SGA group, n/N (%)	1285 (85)	1558/1899 (82)
Trial limitations	Insufficient performance in the standard care group (endotracheal intubation) indicating training issues with the paramedics performing the intervention (supraglottic airway vs. intubation); low rates of initial survival preclude detection of possible neurological outcome effects in survivors	

CPC = cerebral performance category; CPR = cardio-pulmonary resuscitation; ETI = endotracheal intubation; ICU = intensive care unit; SGA = supraglottic airway.

**Table 2:** Patient characteristics in RCTs comparing early compared to late coronary angiography in out-of-hospital cardiac arrest patients with suspected myocardial infarction without ST-segment elevation.

	COACT [10]	TOMAHAWK [11]
Patients, n	552	554
Witnessed arrest, n (%)	421 (81)	462 (83)
Bystander CPR, n (%)	not reported	294 (53)
Shockable rhythm, n (%)	522 (100)	268 (48)
Previous CAD, n (%)	195 (37)	172 (31)
Previous MI, n (%)	149 (27)	89 (16)
Time to ROSC, min		
early CAG, median (IQR)	15 (9–21)	15 (10–20)
late CAG, median (IQR)	15 (8–20)	15 (8–20)
Lactate, mmol/L		
early CAG, median (IQR)	5.3 (3.0–8.8)	5.0 (2.4–8.1)
late CAG, median (IQR)	4.9 (2.8–8.1)	4.9 (2.9–8.4)
Troponin T, ng/L		
early CAG, median (IQR)	44 (29–85)	90 (40–190)
late CAG, median (IQR)	53 (25–116)	80 (40–160)
Revascularization performed		
early CAG, n (%)	107 (39)	93 (37)
late CAG, n (%)	87 (33)	70 (43)
Survival at 90 days,		
early CAG, n (%)	176 (65)	122 (46)
late CAG, n (%)	178 (67)	153 (54)
CPC 1&2 at 90 days		
early CAG, n (%)	133 (52)	91 (36)
late CAG, n (%)	142 (54)	138 (44)
Trial limitations	Low specificity in pre-selection for coronary thrombosis leading to randomization for an intervention not required by the majority of both groups	

CAD = coronary artery disease; CAG = coronary angiography; CPC = cerebral performance category; CPR = cardio-pulmonary resuscitation; IQR = Interquartile Range; MI = myocardial infarction.

**Table 3:** Patient characteristics in RCTs comparing therapeutic hypothermia to controlled normothermia or fever prevention in the 21st century.

	TTM1 [5]	TTM2 [6]
Patients, n	950	1850
Witnessed arrest, n (%)	838 (88)	1702 (92)
Bystander CPR, n (%)	683 (72)	1487 (80)
Shockable rhythm, n (%)	752 (79)	1371 (74)
Time to ROSC, min		
TH, median (IQR)	25 (18–40)	25 (16–40)
Control, median (IQR)	25 (16–40)	25 (17–40)
Time to randomization, min		
TH, median (IQR)	not reported	136 (103–170)
Control, median (IQR)	not reported	133 (99–173)
Lactate, mmol/L		
TH, mean/SD	6.7 ± 4.5	5.9 ± 4.4
Control, mean/SD	6.7 ± 4.5	5.8 ± 4.2
Admission temperature, °C	35.3	35.4
ST-segment elevation, n (%)	384 (40)	749 (40)
Mortality, n (%)	460 (48)	911 (49)
Withdrawal of life support, n (%)	247 (26)	not reported
Prognostication “Poor”, n (%)	not reported	264 (14)
Trial limitations	Increased event rates in both groups following withdrawal of life-sustaining therapy in the absence of negative prognostication (more than 100 patients were terminated for neurological reasons with less than 2 negative prognostication criteria present); delaying the randomized intervention to a point in time when its efficacy is limited (achieving 33 °C only 5–7 h after randomization on average while claiming to do so within 90 min by protocol).	

CPR = cardio-pulmonary resuscitation; IQR = Interquartile Range; TH = therapeutic hypothermia.

**Table 4:** Criteria for and strength of neurological prognostication as applied in large randomized controlled trials, as well as in national and international guidelines.

	TTM1 Trial [5]	TTM2 Trial [6]	ERC European Resuscitation Council [14] *	DGN German Society of Neurology [50]
Date of publication	2013	2021	2025	2023
Strength of prediction	Unknown	Likely poor	Likely poor	Futile
Time of prognostication	≥72 h after the intervention if still unconscious	≥96 h after randomization	≥72 h after ROSC if still comatose and motor score ≤ 3	≥72 h after ROSC if still comatose and motor score ≤ 3
Minimum number of pathological criteria	-	2	2	3
Criteria indicating poor prognosis	persisting coma with a Glasgow Motor Score 1–2 and a treatment refractory status epilepticus, or bilateral absence of N20-peak on median nerve SSEP (for patients in hospitals without SSEP withdrawal of intensive care could be considered if GCS-Motor score did not improve; SSEP only performed in 62% of prognosticated patients)	96 h FOURscore motor response 0–1 and bilateral absence of pupillary and corneal reflexes highly malignant EEG diffuse and extensive anoxic injury on brain CT/MRI Bilaterally absent SSEP N20 responses Serial serum NSE samples consistently higher than locally established levels associated with a poor outcome	no pupillary and corneal reflexes bilaterally absent SSEP N20 responses highly malignant EEG - suppressed background, or - burst suppression status myoclonus diffuse and extensive anoxic injury on brain CT/MRI NSE > 60 µg/L	no pupillary and corneal reflexes bilaterally absent SSEP N20 responses highly malignant EEG diffuse and extensive anoxic injury on brain CT/MRI NSE > 90 µg/L

* in cooperation with the European Society of Intensive Care Medicine (ESICM).

**Table 5:** Evidence supporting de-escalation of guideline recommendations in post-resuscitation care as based on large RCTs.

	Supraglottic Airway Devices	Coronary Angiography in Suspected Coronary Cause	Therapeutic Hypothermia
Setting	OHCA, paramedic-based EMS	NSTE-OHCA, all [11] or only shockable [10] rhythms	2025
Intervention	laryngeal tube [1]/i-gel [2] vs. endotracheal intubation	early vs. deferred coronary angiography	36 °C [5]/normothermia [6] vs. hypothermia 33 °C
Primary endpoint	72 hrs’ survival [1] 30 days’ mRS 0–3 [2]	90 days’ survival [10] 90 days’ mortality [11]	6 months’ mortality
Results	18.3% vs. 15.4%, *p* = 0.04 [1] 6.4% vs. 6.8%, *p* = 0.33 [2]	64.5% vs. 67.2%, *p* = 0.51 [10] 54.0% vs. 46.0%, *p* = 0.06 [11]	48% vs. 50%, *p* = 0.51 [5] 48% vs. 50%, *p* = 0.37 [6]
Conclusion	Among adults with OHCA, initial laryngeal tube insertion was associated with greater 72 h survival compared to initial endotracheal intubation in one trial [1]; supraglottic airway device compared with tracheal intubation did not result in a favorable functional outcome at 30 days in the other trial [2].	Among patients with resuscitated OHCA without signs of STEMI, a strategy of immediate angiography provided no benefit over delayed angiography.	In OHCA patients, hypothermia did not confer a benefit compared to 36 °C or targeted normothermia.
Guideline adoption	Supraglottic airway devices were recommended for all rescuers not highly experienced with endotracheal intubation	Cardiac catheterization delayed if clinical context does not clearly indicate a high likelihood of acute coronary occlusion	Fever prevention recommended instead of therapeutic hypothermia
Potential confounding issues	Long-term outcome may not be interpretable when most patients do not even reach the hospital alive.	It remains unclear how better pre-selection for high likelihood of coronary occlusion can be achieved.	Timely hypothermia was only applied to the minority of patients in the control group and high rate of WLST without minimum prognostic criteria reached.

EMS—emergency medical system; OHCA—out-of-hospital cardiac arrest; NSTE—non-ST-elevation; STEMI—ST-elevation myocardial infarction; WLST—withdrawal of life-sustaining therapy.

## Data Availability

Not applicable.

## Abbreviations

The following abbreviations are used in this manuscript:

CPC	Cerebral performance category
CPR	Cardio-pulmonary resuscitation
ECG	Electrocardiogram
HACORE	HAnnover COoling REgistry
HaCRA	Hannover Cardiac Resuscitation Algorithm
ICU	Intensive care unit
NSTE	Non-ST-segment elevation
OHCA	Out-of-hospital cardiac arrest
RCT	Randomized controlled trials
ROSC	Return of spontaneous circulation
TTM	Targeted temperature management
